# Comparative study of thermal gelation properties and molecular forces of actomyosin extracted from normal and pale, soft and exudative-like chicken breast meat

**DOI:** 10.5713/ajas.18.0389

**Published:** 2018-09-13

**Authors:** Ke Li, Jun-Ya Liu, Lei Fu, Ying-Ying Zhao, Yan-Hong Bai

**Affiliations:** 1 College of Food and Bioengineering, Zhengzhou University of Light Industry, Henan Collaborative Innovation Center for Food Production and Safety, Henan Key Laboratory of Cold Chain Food Quality and Safety Control, Zhengzhou 450001, China

**Keywords:** Pale, Soft and Exudative (PSE)-like, Chicken, Actomyosin, Gel Properties, Molecular Forces

## Abstract

**Objective:**

The objectives of this study were to investigate the thermal gelation properties and molecular forces of actomyosin extracted from two classes of chicken breast meat qualities (normal and pale, soft and exudative [PSE]-like) during heating process to further improve the understanding of the variations of functional properties between normal and PSE-like chicken breast meat.

**Methods:**

Actomyosin was extracted from normal and PSE-like chicken breast meat and the gel strength, water-holding capacity (WHC), protein loss, particle size and distribution, dynamic rheology and protein thermal stability were determined, then turbidity, active sulfhydryl group contents, hydrophobicity and molecular forces during thermal-induced gelling formation were comparatively studied.

**Results:**

Sodium dodecyl sulphate-polyacrylamide gel electrophoresis showed that protein profiles of actomyosin extracted from normal and PSE-like meat were not significantly different (p>0.05). Compared with normal actomyosin, PSE-like actomyosin had lower gel strength, WHC, particle size, less protein content involved in thermal gelation forming (p<0.05), and reduced onset temperature (T_o_), thermal transition temperature (T_d_), storage modulus (G′) and loss modulus (G″). The turbidity, reactive sulfhydryl group of PSE-like actomyosin were higher when heated from 40°C to 60°C. Further heating to 80°C had lower transition from reactive sulfhydryl group into a disulfide bond and surface hydrophobicity. Molecular forces showed that hydrophobic interaction was the main force for heat-induced gel formation while both ionic and hydrogen bonds were different significantly between normal and PSE-like actomyosin (p<0.05).

**Conclusion:**

These changes in chemical groups and inter-molecular bonds affected protein-protein interaction and protein-water interaction and contributed to the inferior thermal gelation properties of PSE-like meat.

## INTRODUCTION

Pale, soft and exudative (PSE)-like poultry meat continues to occur at a high incidence and is a main quality defect in commercial poultry plants, which results in great economic losses to the poultry industry [1]. This meat quality defect affects the sensory quality and consumer’s purchase intentions, and heat-induced gelation ability, water-holding capacity (WHC), emulsion capability, consequently textural characteristic and cooking yield when used in further processed poultry meat products [2]. Petracci et al [3] pointed out that it has become one of large challenges in the research of functional properties of meat proteins for the poultry industry worldwide. These inferior functionalities of meat products are closely related to the denatured state and structural changes of muscle proteins [4]. Previous studies usually focused on the frequency of PSE-like occurrence and evaluated the differences in the solubility of salt soluble proteins between normal and PSE-like meat to indicate the level of denaturation of PSE-like meat proteins [1,5]. Barbut [6] investigating the effects of different chicken meat qualities on the extractable proteins, found that PSE-like meat had lower salt soluble proteins than that of normal meat, and the 151-kDa band was not observed by sodium dodecyl sulphate-polyacrylamide gel electrophoresis (SDS-PAGE), suggesting the lower functionalities of PSE-like meat was attributed to the excessive denaturation of myofibrillar proteins. It is well known that myofibrillar proteins, mainly containing myosin and actin, are the major proteins responsible for thermal gelling formation, which play an important role in texture and WHC of meat products [7]. The dominant myofibrillar proteins are in the form of actomyosin for post-rigor muscles [8]. The physicochemical properties and mechanisms of thermally-induced gelation of myofibrillar proteins extracted from normal meat have been clearly demonstrated [7,9]. However, little information is available concerning the denatured state and thermal gelling formation of PSE-like poultry meat proteins. Only few studies reported the phyco-chemical changes in salt soluble proteins or actomyosin extracted from PSE pork [10, 11]. The basic knowledge of the impaired performance of PSE-like poultry meat proteins are not clearly understood.

In general, many studies concerned salt soluble proteins extraction [3,5]. The reasons for unsuitable gelling properties of proteins from PSE-like meat were mostly explained based on the lower solubility of proteins. The functional properties of proteins in PSE-like meat have not been studied sufficiently for understanding of the factors that influence the functional characteristics of PSE-like meat. It was usually considered that lower protein solubility or less protein extraction were induced by faster glycolysis rate and lower pH of PSE-like meat during rigor development or ionic strength conditions [1,12]. According to our previous studies [5,12], the major factors leading to PSE-like meat having poor protein functionalities are concluded as protein conformation, characteristic of salt soluble proteins, myosin and actin denaturation during meat processing, which also depend on pH and ionic strength conditions. In addition, a three-dimensional network gel is formed by the cross-linking of proteins during heating process, which is based on the strength of different molecular forces such as ionic bonds, hydrogen bonds, hydrophobic interactions [13,14]. Ni et al [15] reported that different pH values affected the charge quantity and regulated the main molecular forces during heat-induced gels formation. A further understanding of changes in the molecular forces of PSE-like chicken meat intrinsic proteins during heating process could be useful to explain the mechanisms in the impaired functional properties of PSE-like chicken meat. Importantly, it is necessary to extract major proteins (actomyosin) and adjust the unified protein concentration, pH and other external factors to compare the molecular functionality and enable an in-depth understanding of the factors that influence the functional characteristics of PSE-like meat.

Therefore, the objectives of this study were to: i) evaluate the thermal gelation properties and level of thermal protein denaturation of actomyosin extracted from normal and PSE-like chicken breast meat, and ii) investigate changes in the molecular forces during thermal-induced gelling formation to further improve the understanding of the variations of functional properties between normal and PSE-like chicken breast meat.

## MATERIALS AND METHODS

### Chicken breast meat selection

Broiler chicken breast (*M. Pectoralis major*) muscles were obtained from a commercial meat plant (Liu-he Group, Tai-an, Shan-dong, China). The average Arbor Acre broilers’ age was 45.2±2.2 days and the average body weight was 2.19±0.41 kg. Briefly, selection and classification of normal and PSE-like chicken meat (a total of 60 normal and PSE-like chicken meat that were collected on three different occasions) using color and pH measurements were according to Li et al [12]. The criteria values for PSE-like meat (L*>53, pH_24 h_<5.7) and normal meat (46<L*<53, 5.7<H_24 h_<6.1) were based on the lightness (L*) and pH value as reported by Li et al [12]. After selection, all skin, fat and the visible connective tissue was trimmed off the chicken breast meat. Then, all samples within each group were cut into cubes (about 1 cm×1 cm×1 cm) and mixed, then tagged, put in plastic bags and frozen (−20°C), and were utilized within two weeks.

### Meat quality measurements

Briefly, the L* values of each chicken breast meat were determined in triplicate using a chromameter (Minolta Camera Co., Osaka, Japan) as described by Li et al [5]. The pH values were measured by inserting an electrode of a portable pH meter (Orion 3-star, Dallas, TX, USA) at three different locations of each breast meat [12]. Drip loss of meat was evaluated at 24 h postmortem. Each breast meat (about 20 g) was placed in a sealed polyethylene package and kept at 4°C for 24 h. Drip loss was expressed as a percentage of the weight loss over initial meat sample weight.

### Extraction of actomyosin

Actomyosin was extracted from both normal and PSE-like meat according to the method of Ogawa et al [16] with minor modifications. Normal and PSE-like meat were thawed at 0°C to 4°C for 24 h prior to extraction. The thawed meat samples were ground three times in a Waring Blender (GM200, Restch, Haan, Germany) at 3,000 r/min for 10 s. The ground samples (approximately 80 g) were suspended in 300 mL of ice-cold isolation buffer (50 mM KCl, 20 mM K_2_HPO_4_/KH_2_PO_4_, pH 7.0), homogenized (Ultraturrax T25, IKA, Staufen, Germany) two times for 30 s at 10,000 r/min and were filtered through a 20-mesh sieve (aperture 0.9 mm). The suspension was centrifuged (Beckman Avanti J-E, Beckman Coulter, Miami, FL, USA) at 10,000 g for 5 min at 4°C. The pellet was collected. These procedures were repeated once. Next, the obtained pellet was homogenized two times for 30 s at 10,000 r/min in 300 mL of chilled 0.6 M KCl buffer (0.6 M KCl, 20 mM K_2_HPO_4_/KH_2_PO_4_, pH 7.0). The suspension was centrifuged at 10,000 for 5 min. The supernatant was filtered through a 20-mesh sieve. Then, the supernatant was blended in 1.2 L of 20 mM potassium phosphate buffer (20 mM K_2_HPO_4_/KH_2_PO_4_, pH 7.0). The precipitate was collected by centrifugation at 10,000 g×10 min and then suspended in 150 mL of ice-cold isolation buffer (50 mM KCl, 20 mM K_2_HPO_4_/KH_2_PO_4_, pH 7.0). The washed precipitate was collected by centrifugation at 10,000 g for 10 min. All procedures were carried out at 4°C. The pellet from the final centrifugation step was collected as actomyosin. The actomyosin concentration was determined by the Biuret method [17]. Actomyosin was stored at 4°C and used in the succeeding tests.

### Sodium dodecyl sulphate-polyacrylamide gel electrophoresis

Actomyosin extracted from normal and PSE-like meat was diluted to 3 mg/mL with buffer (10% β-mercaptoethanol and 0.001% bromophenol blue, 125 mM Tris, 4% SDS, 20% glycerol). Each sample was mixed well and boiled at 95°C for 3 min. The gel was loaded with 15 μg per well of sample solution. SDS-PAGE was performed using a 10% acrylamide resolving gel and a 4% acrylamide stacking gel according to the method of Laemmli [18]. After electrophoresis, the gel was stained for the proteins using a solution containing 0.1% (w/v) Coomassie brilliant blue R-250, 40% (v/v) ethanol, and 7% (v/v) glacial acetic acid. Subsequent to destaining, the gels were scanned (GT-800F, Epson, Hirookanomura, Japan).

### Particle size and distribution

Particle size distribution measurement was determined according to the method of Li et al [19] with slight modifications. The particle size of actomyosin was determined by static light scattering using a Mastersizer 2000 laser light scattering analyzer (Malvern Instruments Ltd., Malvern, UK). Normal and PSE-like actomyosin were stirred and dispersed in potassium phosphate buffer solution with two different KCl concentrations (50 mM KCl or 0.6 M KCl, 20 mM K_2_HPO_4_/KH_2_PO_4_, pH 7.0), respectively. The particle distribution of each sample was monitored over four successive readings. The particle size was expressed as D_50_, D_90_, D_3,2_, and D_4,3_ as follows:

D_50%_, the size of the particle for which 50% of the sample is below this size;
D_90%_, the size of the particle for which 90% of the sample is below this size;
D_(3,2)_, the surface area moment mean diameter, D_(3,2)_ = ∑n_i_d_i_^3^/∑n_i_d_i_^2^, where n_i_ is the number of particles with diameter d_i_ and was calculated from the size distribution;
D_(4,3)_, the volume moment mean diameter, D_(4,3)_ = ∑n_i_d_i_^4^/ ∑n_i_d_i_^3^, where n_i_ is the number of particles with diameter d_i_ and was calculated from the size distribution.

### Gel strength

Actomyosin extracted from normal and PSE-like meat was adjusted to 40 mg/mL using 0.6 M KCl buffer (0.6 M KCl, 20 mM K_2_HPO_4_/KH_2_PO_4_, pH 7.0). Each sample (40 mg/mL) was added to a 10-mL glass beaker (25 mm diameter, 35 mm height). Then, the beakers were sealed with parafilm, heated and kept in a water bath (Julabo TW20, Gerhard-Juchheim-Strasse, Germany) at 80°C for 20 min. The gels were cooled in an ice bath and stored at 4°C overnight for analysis. The obtained gels were equilibrated to ambient temperature (20°C). The strength of the gels was measured using a Texture Analyzer (TA.XT2i, Stable Micro Systems, Godalming, UK) according to the method of Chen et al [20] with slight modifications. The samples were subjected to a penetration test. P/0.5 probe was employed with 2.0 mm/s pre-test speed, 1.0 mm/s test speed and a 3 g trigger force. The penetration distance was 10 mm. Gel strength was defined as the first peak force.

### Water-holding capacity of actomyosin

The WHC of actomyosin was measured using a centrifugal method as described by Kocher and Foegeding [21] with a minor modification. The actomyosin (approximately 6 g) was added to a 10-mL centrifuge tube, heated and kept in a water bath (Julabo TW20, Germany) at 80°C for 20 min, and then cooled to room temperature. The tubes were centrifuged at 10,000×g (Beckman Avanti J-E, Beckman Coulter, USA) for 10 min. The original weight of actomyosin samples and the weight of the centrifuge tubes were recorded. Finally, the amount of liquid released was collected and measured. The WHC was calculated by the percentage of the amount of released water relative to the original weight of actomyosin samples (g). A low value demonstrates the gel has a superior WHC to the sample with a high value.

### Protein loss

After determining the WHC of the gels, protein loss of each actomyosin gel was measured by the method as described by Camou and Sebranek [10]. A l mL aliquot of the released liquid from the gels was obtained, then the protein concentration was measured by the Biuret method [17].

### Differential scanning calorimetry

Differential scanning calorimeter (Lindon, UT84042, TA Instruments, New Castle, DE, USA) was used to measure the onset (T_o_), maximum (T_d_), and endset (T_e_) temperatures for actomyosin endothermic transitions as well as denaturation enthalpy (ΔH) of actomyosin as described by Li et al [12] with slight modifications. 0.5 g of each sample was accurately weighed and kept at 20°C for 10 min in an aluminum pan. The thermal properties of actomyosin was determined by heating the samples in aluminum pans from 20°C to 85°C at 0.5°C/min. The supplied software (Thermal Analysis System 2000, Version 4.5A, TA Instruments, New Castle, DE, USA) was used to estimate these thermal parameters.

### Dynamic rheological measurements

Dynamic rheological measurements of actomyosin were performed using a rheometer (Physica MCR 301, Anton Paar, Graz, Austria) equipped with a 25 mm parallel plate geometry in the oscillatory mode according to the method of Li et al [5]. The actomyosin (40 mg/mL) samples were placed and tempered between parallel plates with a 1 mm gap. Silicone oil was used to the edge of sample to prevent evaporation. Each sample was kept at 25°C for 3 min, then heated from 25°C to 80°C at a scan rate of 1°C/min. The storage modulus (G′) and loss modulus (G″) of the samples were monitored during dynamic oscillatory measurements. G′ was used to determine the energy stored resulting from elastic deformation of the gel network. G″ performed the viscous property of actomyosin samples during the gelatinization process.

### Turbidity

Turbidity of actomyosin was determined as described by Wang et al [8] with some modifications. Each actomyosin sample was diluted to 2.5 mg/mL using 0.6 M KCl buffer (0.6M KCl, 20 mM K_2_HPO_4_/KH_2_PO_4_, pH 7.0) and measured by a UV/Vis spectrophotometer with a temperature control unit (Shimadzu, Nara-shi, Japan). The 0.6 M KCl buffer was used as a reference. Each sample was heated from 20°C to 85°C at 1°C/min and the absorbance of each samples at 350 and 660 nm was determined and obtained once every 10°C.

### Reactive sulfhydryl group

The reactive sulfhydryl content was measured according to the method of Ellman [22] with slight modifications. Briefly, each actomyosin (normal and PSE-like actomyosin, respectively) was diluted to 2.0 mg/mL using 0.6 M KCl buffer. Then, 2 mL of each actomyosin solution (2 mg/mL) was placed in a 7-mL centrifuge tube and heated in a water bath from 20°C to 85°C at 1°C/min. Twenty-one tubes were placed in water bath (Julabo TW20, Germany) for each actomyosin. Three tubes were taken out every 10°C intervals (total seven sampling points), then transformed in an ice water bath for 10 min. Subsequently, 5 μL of 5-5′-dithiobis-2-nitrobenzoic acid was added to the actomyosin solution and kept at room temperature for 20 min. The absorbance of the actomyosin solution was determined at 412 nm (SpectraMax M_2_, Molecular Devices Limited, Sunnyvale, CA, USA). Reactive sulfhydryl group content was calculated using the extinction coefficient of 13,600 M^−1^ cm^−1^.

### Surface hydrophobicity

The surface hydrophobicity of actomyosin was examined according to the method of Zhao et al [14] with slight modifications. Each actomyosin was diluted to 2.0 mg/mL using 0.6 M KCl buffer. Each actomyosin solution (2 mL) was placed in a 7-mL centrifuge tube and heated in a water bath (Julabo TW20, Germany) from 20°C to 85°C at 1°C/min. Twenty-one tubes were placed in water bath (Julabo TW20, Germany) for each actomyosin. Three tubes were taken out every 10°C intervals (total seven sampling points for each actomyosin), and then transformed in an ice water bath for 10 min. Each actomyosin suspension (2 mL) was mixed with 80 μL of bromophenol blue (1 mg/mL) (BPB). The KCl buffer (0.6 M KCl, 20 mM K_2_HPO_4_/KH_2_PO_4_, pH 7.0, 2 mL) with 80 μL of BPB addition was used as the control. The control and samples were placed at 20°C, mixed for 10 min and centrifuged at 4,000 g for 15 min (Beckman Avanti J-E, Beckman Coulter, USA). After centrifugation, the liquid supernatant was diluted 10 times. The absorbance of the control (A_0_) and the sample (A_s_) were determined at 595 nm. The 0.6 M KCl buffer was used as reference. The BPB bond content was expressed as the following equation: BPB bound (μg) = 80 μg×(A_0_–A_s_)/A_o_.

### Molecular forces

Molecular forces of actomyosin during heating process were determined as described by Ni et al [15]. Each of actomyosin sample (1 mL) was added to 10-mL centrifuge tube. Each tube was heated from 20°C to 85°C at 1°C/min in a water bath (Julabo TW20, Germany). The tube was taken out every 10°C intervals. Seven sampling points including 20°C, 30°C, 40°C, 50°C, 60°C, 70°C, and 80°C were chosen. Eighty-four tubes were used and placed in water bath (Julabo TW20, Germany). Then the tubes were added to 5 mL of four chemical solutions: solution A (SA) 0.05 M NaCl; solution B (SB) 0.6 M NaCl; solution C (SC) 1.5 M urea+0.6 M NaCl; solution D (SD) 8.0 M urea+0.6 M NaCl, which disrupted some bond types including ionic bonding, hydrogen bonding and hydrophobic interactions. Proteins were partially solubilized in these solutions to measure the existence of ionic bonds (difference between SB and SA), hydrogen bonds (difference between SC and SB) and hydrophobic interactions (difference between SD and SC). In total 28 solutions (three tubes for each solution) were obtained and then homogenized (Ultraturrax T_25_, IKA, Germany) two times for 30 s at 10,000 rpm. The mixtures were placed at 4°C for 1 h and centrifuged (Beckman Avanti J-E, Beckman Coulter, USA) at 10,000×g for 15 min. the Biuret method were used to measure the protein concentration of the supernatants [17]. The ionic bonding was expressed as the difference in protein concentrations between SA and SB samples. Hydrogen bonding was expressed as the difference in protein concentrations between SC and SB samples. Hydrophobic interaction was expressed as the difference in protein concentrations between SD and SC samples.

### Statistical analysis

The actomyosin was extracted from normal and PSE-like meat at different occasions to conduct three independent replications. All data were presented as mean±standard deviation. Statistical Analysis System (SAS 8.2) (SAS Institute Inc., Cary, NC, USA) was used to carry out the statistical analysis of the data. The independent sample *t* test was used to compare the difference in meat qualities, gel strength, differential scanning calorimetry (DSC) data, chemical groups and molecule forces between normal and PSE-like actomyosin. The data obtained for the particle size, biochemical changes and molecule forces of actomyosin during heating process were submitted to analysis of variance by the general linear model procedures of SAS 8.2. Duncan’s multiple range test (p<0.05) was applied to compare the means of variables among different treatments.

## RESULTS AND DISCUSSION

### The characteristics of normal and pale, soft and exudative-like chicken breast meat

The characteristics of normal and PSE-like meat are shown in Table 1. Significant differences were observed (p<0.05) for L* and pH at 3 h and 24 h between normal meat and PSE-like meat. The measurement of drip loss clearly indicated that each color group was representative for the two different meat qualities. Therefore, two group chicken breast meat samples were used for this study to further analysis.

### Sodium dodecyl sulphate-polyacrylamide gel electrophoresis of actomyosin

**Figure 1:**
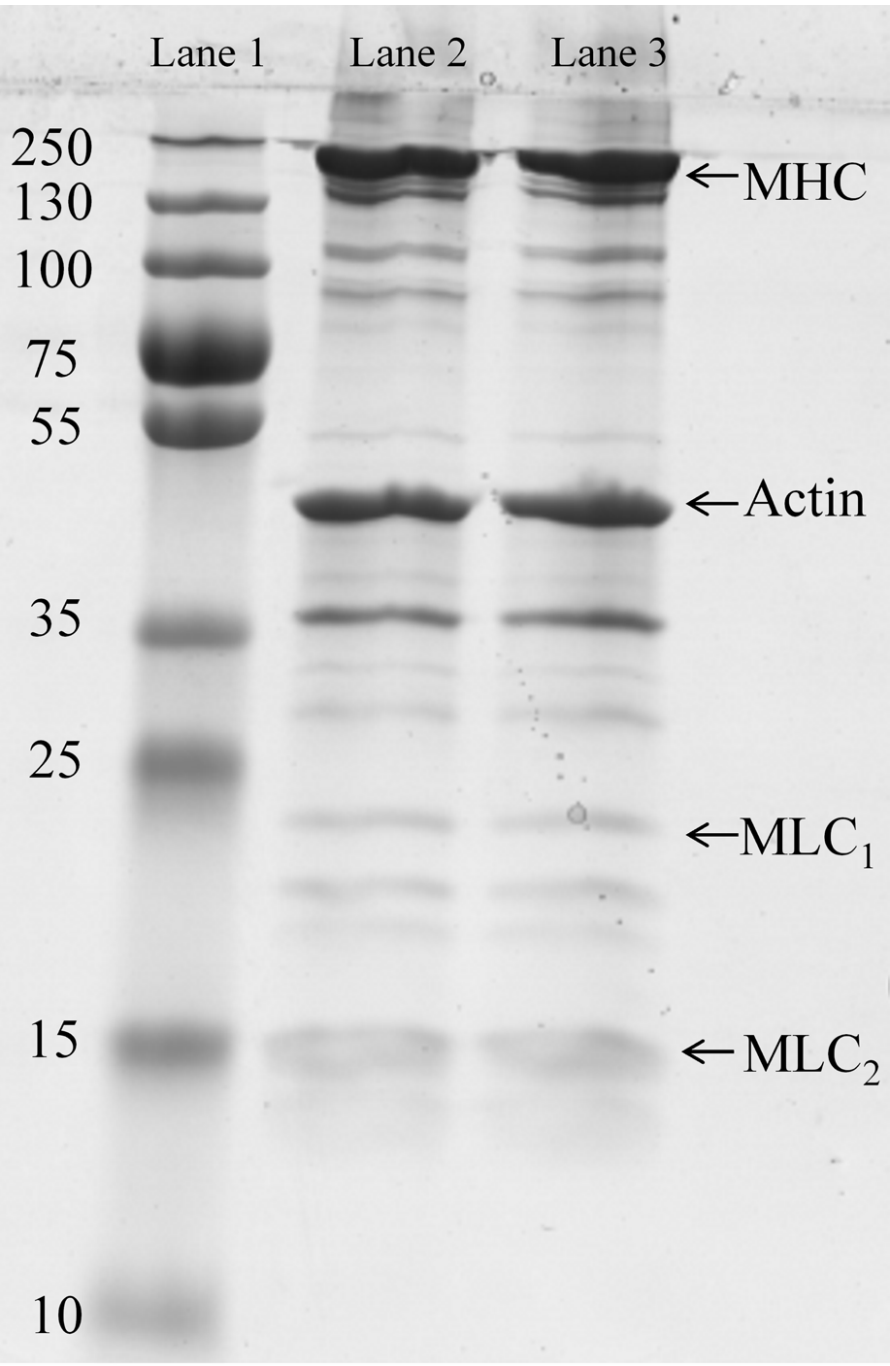
Sodium dodecyl sulphate-polyacrylamide gel electrophoresis of normal and pale, soft and exudative (PSE)-like actomyosin. Lanes 1, standard marker; lanes 2, normal actomyosin; lanes 3, PSE-like actomyosin; MHC, myosin heavy chains; MLC, myosin light chains.

Figure 1 shows the protein profiles for actomyosin extracted from normal and PSE-like meat by SDS-PAGE. There were similar protein profiles between normal and PSE-like actomyosin. These protein profiles are mainly comprised myosin and actin, and containing other proteins such as troponin, tropomyosin, α-actinin and c-protein which are mainly connected with myosin and actin [23]. There were no significant differences (p>0.05) in the characteristics of actomyosin, suggesting that the associated variation in gel properties between normal and PSE-like meat were attributable to other physico-chemical properties instead of the variation in the protein bands of extracted actomyosin. The results agreed with Chan et al [24], who found that electrophoretic band patterns of myosin and actin from PSE-like and normal turkey meat were not different. However, Barbut et al [6] found the loss of 151-kDa band in the proteins extracted from PSE like meat. The different SDS-PAGE results between the present study and Barbut et al [6] may be due to the different samples. The protein samples were extracted from individual broiler breast in the report by Barbut et al [6] while the mixed broiler meat was used for this study. Eadmusik et al [25] found that the different individual turkey breast meat had different protein profiles identified by SDS-PAGE and Western blotting although the protein samples were extracted from the same fast glycolysing group in term of PSE syndrome development.

### Particle size distribution

**Figure 2:**
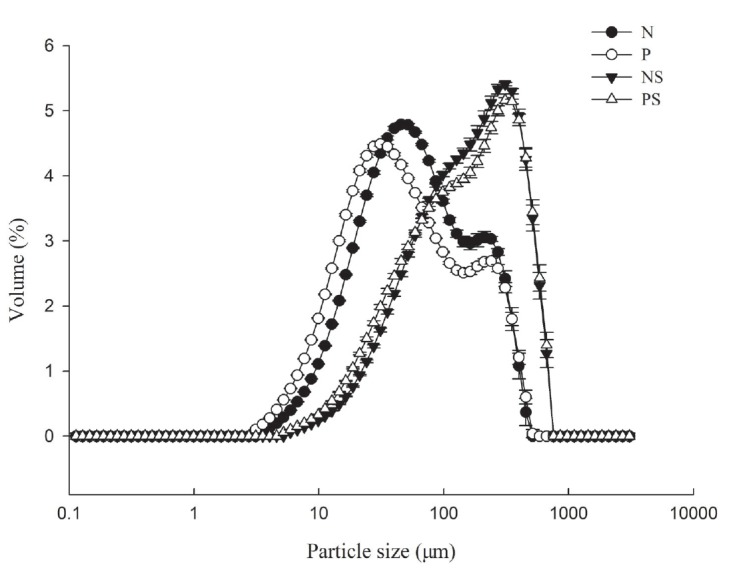
Particle size distribution of normal and pale, soft and exudative (PSE)-like actomyosin under different KCl concentration conditions. N and P, normal and PSE-like actomyosin at low KCl concentration condition (50 mM KCl); NS and PS, normal and PSE-like actomyosin at high KCl concentration condition (0.6 M KCl).

The changes in particle size distribution of normal and PSE-like actomyosin under different KCl concentrations are shown in Figure 2 and Table 2. In low KCl concentration condition (50 mM KCl), the profile of actomyosin in general consisted of two major peaks (one peak range 10 to 100 μm, another peak range 100 to 1,000 μm). In high KCl concentration condition (0.6 mol/L KCl), the particle size distribution profiles of actomyosin became more uniform, occurring a transformation of particle size distribution with a larger number of particles (100 to 1,000 μm). The higher ionic strength increased the charge of actomyosin and affected the electrostatic interactions to cause variable shifting in the particle size distribution [26,27].

There were significant differences in particle size distribution between normal and PSE-like actomyosin (p<0.05) (Table 2). In low salt concentration, PSE-like actomyosin had lower the values of D_50_, D_3,2_, and D_4,3_ than those of normal actomyosin. The high ionic strength caused a significant increase in particle size (D_50_, D_90_, D_3,2_, and D_4,3_) of both normal and PSE-like actomyosin (p<0.05). Compared with normal actomyosin, PSE-like actomyosin had lower values of D_50_ and D_3,2_ (p<0.05). PSE-like actomyosin had lower particle size than that of normal actomyosin, which was attributed to different glycolytic development between PSE and normal meat. Actomyosin is the main existing form of myosin and actin filaments in the rigor state [28]. PSE-like meat had both faster acidification process and extent, thus resulting in a more tightly binding of myosin with actin [12,28]. It is proposed that the particle size variation may induce the difference in gel forming ability between normal and PSE-like meat.

### Gel strength, water-holding capacity, protein loss and differential scanning calorimetry analysis

As shown in Table 3, there were significant difference in gel strength, WHC and protein loss between normal and PSE-like actomyosin (p<0.05). The gel strength and WHC of PSE-like actomyosin were lower than those of normal actomyosin at the same protein concentration. The results indicated that the native proteins from PSE-like meat have less functionalities including the lower thermal gelling ability and less effectively trapping water. Protein loss in the expelled liquid after cooking and centrifugation for the PSE-like actomyosin were significantly higher than that for normal actomyosin (p<0.05), suggesting that less protein-protein interaction occurs in the PSE-like meat during heating process. Camou and Sebranek [10] also found that there were similar observations in salt soluble proteins of PSE pork. These results indicated that the associated functional variation in PSE-like meat could not be explained solely by the variation in the salt soluble protein extraction and pH of PSE-like meat when used in common further processing. Consequently, changes in characteristics of the intrinsic muscle proteins were possibly an important factor in causing weaker thermal gelation ability and WHC of PSE-like meat. Table 3 also shows the thermal denaturation temperature and enthalpy in normal and PSE-like actomyosin. Compared with normal actomyosin, there were significant reduction in T_o_ and T_d_ from PSE-like actomyosin (p<0.05). This indicated that actomyosin extracted from PSE-like meat were destabilized and became more susceptible to thermal denaturation. However, no significant difference was observed in T_e_ and ΔH between normal and PSE-like actomyosin (p>0.05).

Previous studies reported thermal denaturation of muscle proteins extracted from different meat qualities [29,30]. Liu et al [30] reported that there were three or five endothermic transition peaks for chicken myofibrillar proteins after the separation and purifying. Park and Lanier [31] found that there were three endothermic transition peaks for tilapia proteins after purification, mainly corresponding to myofibril (47.8°C), myosin (57.7°C), and actin (68°C). These transition temperatures indicated proteins conformational changes during thermal denaturation, one of which was transformed from α-helix into random coil for promoting the thermal induced gelation of myofibrillar proteins. In addition, the range of transition temperature for myosin and actomyosin were very wide for different studies [30,31]. The denatured temperature range for the purified myosin and actin were from 39.6°C to 65.1°C and 43.5°C to 71.1°C, respectively. It was suggested that the extracted protein denaturation temperature probably have different values, which may be due to specific pH and ionic conditions, use of different proteins extraction methods or the sample concentrations [32,33]. Wright et al [33] reported that no endothermic transition peaks were observed when the concentration of protein was adjusted to higher than 20 mg/mL, because proteins formed a gel network during heating. The present study found one endothermic transition peak from 50°C to 55°C, indicating an increase binding of actomyosin. Actin could interact with myosin during thermal treatment, thus resulted in the changes of gel elasticity [12]. PSE-like actomyosin exhibited lower thermal stability of actomyosin suggesting there is a variation in the denaturation and aggregation of actomyosin to induce the differences in the gel properties between normal and PSE-like actomyosin.

The lower gel strength and WHC of PSE-like actomyosin probably resulted from the faster acidification rate and higher acidification extent in term of PSE-like syndrome development [12]. Rapid glycolysis or lower pH in PSE-like meat induced changes in the myofibrillar structures, promoted higher protein denaturation and degradation of proteins [34,35], which may lower the transition temperature and limit the gel-forming ability of actomyosin, thus have a negative effect on the gel strength and WHC. The higher rate and extent of protein degradation in PSE-like meat also resulted in the smaller particle size of PSE-like actomyosin [36], which may lead to the differences (low gel strength, low WHC, low heat stability) between actomyosins extracted from normal and PSE like meat.

### Storage modulus and loss modulus

**Figure 3:**
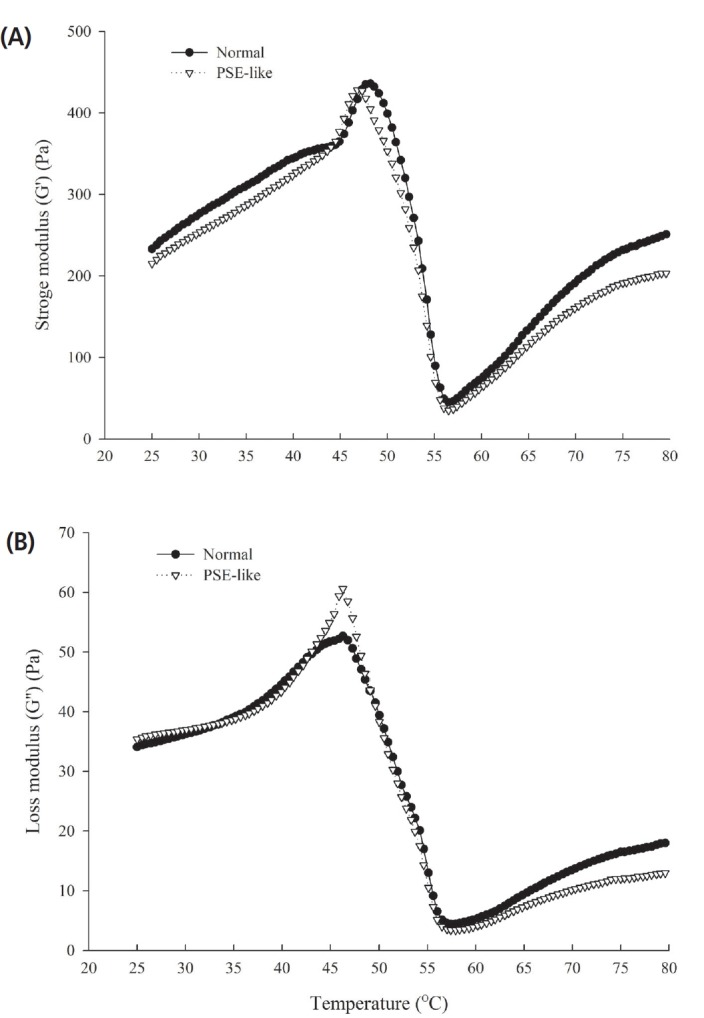
Changes in dynamic storage modulus (G′) (A) and loss modulus (G″) (B) during heating from 25°C to 80°C for normal and pale, soft and exudative (PSE)-like actomyosin.

Figure 3 shows changes in G′ and G″ of normal and PSE-like actomyosin during heat-induced gelation process. The G′ of normal actomyosin increased slowly from heating to approximately 45°C in the beginning, then increased quickly to a maximum at 49°C with exhibiting a peak (Figure 3A). It then decreased sharply to a minimum at 56°C. On further heating to 80°C, the G′ increased steadily. The early increase in G′ indicated that the preliminary elastic protein network structure, which was mainly due to the unfolding and denaturation of myosin heads [37]. The rapid increase of G′ was closely related to the unfolding and cross-linking of myosin tails [37]. Then, denaturation of the myosin light chain induced G′ to decease sharply and increased the mobility of muscle proteins [38]. On subsequent heating, the increase of G*′* was attributed to an increase in the amounts of actomyosin cross-links between protein aggregates/strands [39]. The G′ of PSE-like actomyosin was lower than that of normal actomyosin at first from heating to approximately 44°C, then increased sharply up to maximum at 47°C. It was obvious that a peak earlier appeared for PSE-like actomyosin (Figure 3A). The different change in G′ in PSE-like actomyosin may be due to formation of more protein interactions and aggregates at lower temperature. Subsequently, the G′ of PSE-like actomyosin increased relatively slow on further heating and was lower than that of normal actomyosin, indicating heat-induced PSE-like actomyosin gel was less rigid and elastic. These corresponded to the results of the gelation strength of actomyosin. Wang et al [8] reported that the G′ of actomyosin extracted from normal and PSE pork have different rheological transitions. The dynamic rheological behavior reflected the different changes in biochemical properties of PSE-like native protein during heating, which inferred that it is an important factor affecting the gelation properties of PSE-like chicken meat in addition to the different extents of protein solubility and their pH values.

Figure 3B shows the changes of G″ for normal and PSE-like actomyosin during heating process. The first peak of both normal and PSE-like actomyosin appeared at 46°C, then the G″ decreased rapidly to a minimum at 57°C. Then the changes in G″ for both normal and PSE-like actomyosin were like those observed for the G′. The G″ value at the end heating points indicated that PSE-like actomyosin reduced the viscosity compared to normal actomyosin.

### Turbidity, reactive sulfhydryl and surface hydrophobicity of actomyosin

**Figure 4:**
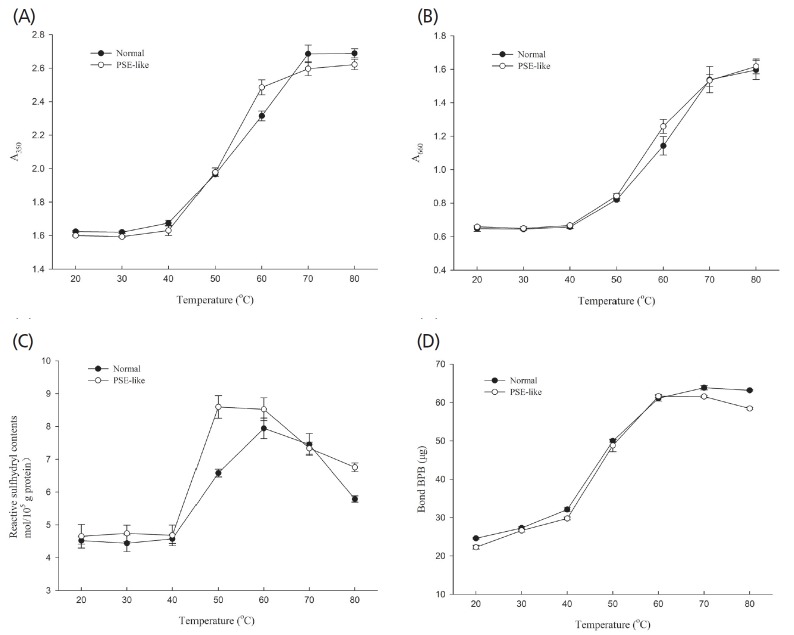
Changes in turbidity measured at 350 nm (A) and 660 nm (B), reactive sulfhydryl (C) and surface hydrophobicity (D) of normal and pale, soft and exudative (PSE)-like actomyosin during heating.

Figure 4 shows changes in turbidity, reactive sulfhydryl and surface hydrophobicity of normal and PSE-like actomyosin when temperature rose from 20°C to 80°C. As shown in Figure 4A and 4B, both normal and PSE-like actomyosin became more turbid as temperature rose, suggesting protein aggregates were formed and increased. Turbidity was a good indicator of the extent of protein aggregation [40]. The turbidity of normal and PSE-like actomyosin slightly increased during heating from 20°C to 40°C. The turbidity values did not differ significantly during this temperature range. The turbidity was increased significantly from 40°C to 60°C (p<0.05). The rate of turbidity for PSE-like actomyosin was higher than that for normal actomyosin during heating from 40°C to 60°C. There was a significant difference at 60°C in the turbidity values (p< 0.05) between normal and PSE-like actomyosin (Figure 4A, 4B), indicating the denaturation and aggregation of PSE-like actomyosin were more intense during this temperature range. On further heating from 60°C to 80°C, the turbidity for normal and PSE-like actomyosin continued to go up (Figure 4A; 4B). There were significant differences in the turbidity between PSE-like actomyosin normal actomyosin (p<0.05) in the range 70°C to 80°C (Figure 4A). This indicated the protein aggregates of PSE-like actomyosin were smaller than those of normal actomyosin. The protein aggregates or large particles were formed by heavy meromyosin and light meromyosin via changing their conformations [37]. Chan and Gill [41] reported that the differences in turbidity were most probably attributed to the variation in the size and/or rate of protein aggregation. These results suggested the varying size and rate of protein aggregation may induce the difference in the gel strength between normal and PSE-like actomyosin.

As shown in Figure 4C, the reactive sulfhydryl of normal and PSE-like actomyosin did not differ significantly during heating from 20°C to 40°C. The reactive sulfhydryl values increased significantly from 40°C to 60°C (p<0.05), indicating actomyosin was unfolded to form inter-intra-molecular interactions that could induce protein aggregation [9]. The reactive sulfhydryl value was higher in the PSE-like actomyosin compared to normal actomyosin, suggesting PSE-like actomyosin exhibited a rapid unfolding rate and exposed the buried sulfhydryl groups to its surface [11,12]. On further heating from 60°C to 80°C, the reactive sulfhydryl value was significantly decreased (p<0.05), suggesting the formation of disulfide linkage. PSE-like actomyosin had higher reactive sulfhydryl value than normal actomyosin (p<0.05) at 80°C. Compared to normal actomyosin, PSE-like actomyosin began to unfold rapidly at 40°C and had a higher temporal rise of reactive sulfhydryl while the decrease of reactive sulfhydryl converted less effectively to disulfide bond on further heating. Lanier [42] reported that the exposed reactive sulfhydryl groups which tend to form the formation of disulfide linkages, played an important role in the gel properties. These differences in reactive sulfhydryl contents confirmed that PSE-like actomyosin had less protein interactions resulting in the increased protein loss and decreased gel strength than normal actomyosin.

As shown in Figure 4D, the surface hydrophobicity of normal and PSE-like actomyosin slightly increased during heating from 20°C to 40°C. The surface hydrophobicity of PSE-like actomyosin was significantly lower (p<0.05) than that of normal actomyosin. Then a significant increase of surface hydrophobicity occurred for both normal and PSE-like actomyosin as temperature rose from 40°C to 60°C (p<0.05). This result indicated the changes in conformation of actomyosin including exposing hydrophobic aromatic amino acid residues to protein surface, which induced the formation of hydrophobic interaction to participate in the gelation process [43,44]. There was no significant difference between the surface hydrophobicity of normal and PSE-like actomyosin at 50°C and 60°C (p>0.05), suggesting PSE-like actomyosin exhibited a rapid unfolding rate and exposed the buried hydrophobic aromatic amino acid residues to its surface during heating from 20°C to 60°C [45]. On subsequent heating to 80°C, the surface hydrophobicity of PSE-like actomyosin was significantly lower (p<0.05) than that of normal actomyosin. Sankar and Ramachandran [46] reported that the hydrophobic groups becoming more exposed contributed to the formation of insoluble, turbid aggregates. These results indicated that the difference in the hydrophobic groups could explain the different gelation properties of actomyosin.

### Molecular forces

**Figure 5:**
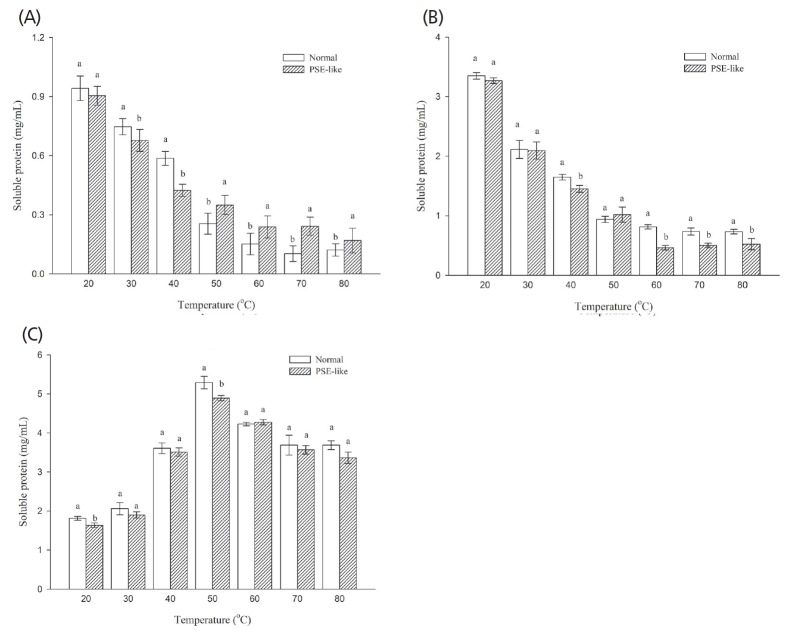
Changes in ionic bond (A), hydrogen bond (B), and hydrophobic interaction (C) of normal and pale, soft and exudative (PSE)-like actomyosin during heating. ^a–b^ Different letters indicate significant differences among the means at the same temperature.

Different molecular forces of protein-protein interactions during heating process contributed to form and maintain the gelation structures, which included ionic bonds, hydrogen bonds and hydrophobic interactions [14]. Thus, changes in these molecular forces involved in the gelling formation influenced the gel properties of muscle proteins. Different chemical solutions (SA, SB, SC, and SD as aforementioned in section Methods) were chosen to disrupt some types of intermolecular bonds to further understand the variation in the gel formation of normal and PSE-like actomyosin. As shown in Figure 5, normal and PSE-like actomyosin showed similar patterns of molecular forces during heating, as has also been shown previously [47]. The protein solubility as expressed the individual molecular interaction was affected significantly by heating (p<0.05). There was a significant decrease (p<0.05) in the ionic and hydrogen bonds as the temperature increased, indicating that the ionic and hydrogen bonds were broken. The ionic bond of PSE-like actomyosin was slightly lower (p>0.05) than that of normal actomyosin at 20°C. When heating to 40°C, the ionic bond of PSE-like actomyosin was significantly lower (p<0.05) than that of normal actomyosin. On further heating from 50°C to 80°C, the ionic bond of PSE-like actomyosin was significantly higher (p<0.05) than that of normal actomyosin, while the hydrogen bond of PSE actomyosin was lower significantly than that of normal actomyosin (p<0.05) on heating from 60°C to 80°C. Hydrophobic interaction started to increase at 20°C and reached a maximum at 50°C, then gradually fell to a steady state on further heating. Heating induced the unfolding of the peptide chains in native proteins and increased the exposure of some buried hydrophobic groups to extend on the surfaces [15], which was related to changes in the conformation of myosin tails. Compared to the ionic and hydrogen bonds, the value of protein solubility as expressed by the hydrophobic action was the highest (p<0.05) at the end of heating treatment, which indicated that the main molecular force was hydrophobic interaction contributing to the gelation properties of proteins. However, the hydrophobic interaction of PSE-like actomyosin was not significantly lower than that of normal actomyosin (p>0.05) on heating from 60°C to 80°C. The significant differences in the ionic and hydrogen bonds were observed between normal and PSE-like actomyosin at the end of heating treatment (p<0.05), which may induce the difference in gelation properties between normal and PSE-like actomyosin. The gel network formation of muscle proteins was constructed and maintained by these molecular interactions of protein-protein and protein-water in the gel system [48]. The contribution of various chemical forces to maintain the gel network varies in different protein systems and conditions. Liu et al [13] reported that ionic bonds and hydrogen bonds contributed significantly to stabilize protein conformations, increase protein- water interactions and gel formation of muscle proteins.

## CONCLUSION

The experiments performed in this study investigated the thermal gelling properties and molecular forces of actomyosin extracted from normal and PSE-like chicken breast meat. PSE-like actomyosin had lower gel strength, WHC and less protein content involved in the thermal gelling forming. SDS-PAGE showed similar patterns of actomyosin extracted from PSE-like and normal meat. However, PSE-like actomyosin had different viscoelastic patterns of thermal gelation from normal actomyosin and reduced the G′ and G″. The transition temperature of G′, particle size and the thermal transition temperature (T_d_) for PSE-like actomyosin were lowered than those for normal actomyosin. Moreover, the rate of increase was higher for the turbidity, sulfhydryl group contents and hydrophobicity of PSE-like actomyosin in the range of 40°C to 60°C, indicating that protein aggregation was more intense. While the transition from reactive sulfhydryl group into a disulfide bond of PSE-like actomyosin was less than those normal actomyosin after heating treatment. Molecular forces showed the different changes in ionic and hydrogen bonds between PSE-like and normal actomyosin at the end of heating treatment, indicating these differences in the formation of chemical groups and intermolecular bonds possibly affected protein-protein interaction and contributed to diverse gelation properties between PSE-like and normal actomyosin. This study clearly demonstrated the loss in native protein functionality caused the impaired functional properties of PSE-like chicken breast meat besides other factors such as pH and lower protein extraction during meat traditional processing.

## Figures and Tables

**Table 1:** Characteristics of normal and PSE-like chicken meat

Parameter1)	Meat color group	

	Normal	PSE-like
L*_3 h_	49.79±2.24^b^	55.11±2.08^a^
pH_3 h_	6.02±0.06^a^	5.85±0.07^b^
L*_24 h_	51.86±1.42^b^	58.63±2.21^a^
pH_24 h_	5.98±0.07^a^	5.63±0.05^b^
Drip loss (%) _24 h_	1.36±0.51^b^	3.59±1.12^a^

PSE, pale, soft and exudative.

1) L*_3 h_ and pH_3 h_ were determined at 3 h postmortem; L*_24 h_ and pH_24 h_ were determined at 24 h postmortem; Drip loss (%)_24 h_ was determined at 24 h postmortem.

a–b Different letters indicate significant differences between the means in the same row (p<0.05).

**Table 2:** Particle size of normal and PSE-like actomyosin under different KCl concentration conditions

Samples2)	Particle size1) (μm)			

	D_50_	D_90_	D_3,2_	D_4,3_
N	60.30±0.40^c^	256.00±7.94^b^	38.10±0.20^c^	99.90±2.02^b^
P	47.40±0.40^d^	254.00±5.86^b^	29.80±0.15^d^	91.80±1.51^c^
NS	171.00±3.21^a^	474.00±13.05^a^	89.30±1.04^a^	218.00±5.29^a^
PS	161.00±3.00^b^	481.00±12.22^a^	78.30±0.95^b^	215.00±5.03^a^

PSE, pale, soft and exudative.

1) D_50%_, the size of the particle for which 50% of the sample is below this size; D_90%_, the size of the particle for which 90% of the sample is below this size; D_(3,2)_, the surface area moment mean diameter, D_(3,2)_ = ∑n_i_d_i_^3^/∑n_i_d_i_^2^, where ni is the number of particles with diameter di and was calculated from the size distribution; D_(4,3)_, the volume moment mean diameter, D_(4,3)_ = ∑n_i_d_i_^4^/∑n_i_d_i_^3^, where n_i_ is the number of particles with diameter d_i_ and was calculated from the size distribution.

2) N and P, normal and PSE-like at low KCl concentration condition (50 mM KCl); NS and PS, normal and PSE-like actomyosin at high KCl concentration condition (0.6 M KCl).

a–d Different letters indicate significant differences among the means in the same column (p<0.05).

**Table 3:** Gel strength, WHC, protein loss, thermal denaturation temperature and enthalpy of normal and PSE-like actomyosin

Parameter	Actomyosin	

	Normal	PSE-like
Gel strength (g)	32.60±3.29^a^	26.41±1.98^b^
WHC (%)	31.95±3.06^b^	37.04±4.22^a^
Proteins loss (mg/mL)	2.74±0.18^b^	3.15±0.26^a^
DSC		
T_o_ (°C)	53.29±0.65^a^	50.01±0.53^b^
T_d_ (°C)	55.95±0.46^b^	53.58±0.25^a^
T_e_ (°C)	57.93±1.09^a^	57.45±1.07^a^
ΔH (J/g)	0.021±0.002^a^	0.020±0.001^a^

WHC, water-holding capacity; PSE, pale, soft and exudative; DSC, differential scanning calorimetry; T_o_, the onset temperature of thermal denaturation for actomyosin; T_d_, the maximum temperature of thermal denaturation for actomyosin; T_e_, the end temperature of thermal denaturation for actomyosin; ΔH, the enthalpy of thermal denaturation for actomyosin.

a–b Different letters indicate significant differences between the means in the same row (p<0.05).
